# Work-related mental disorders: the interface between occupational factors and alcohol and other drug misuse

**DOI:** 10.47626/1679-4435-2026-1602

**Published:** 2026-07-26

**Authors:** Eduardo William Farinha Carvalho, Veronica Perius de Brito, Alice Mirane Malta Carrijo, Marcos Vinicius Teixeira Martins, Stefan Vilges de Oliveira

**Affiliations:** 1 Universidade Federal de Uberlândia, Curso de Graduação em Medicina, Uberlândia, MG, Brazil; 2 Universidade Federal de Uberlândia, Departamento de Saúde Coletiva, Uberlândia, MG, Brazil

**Keywords:** mental disorders, substance-related disorders, alcohol consumption, epidemiology, occupational health

## Abstract

**Introduction:**

Work-related mental disorders comprise conditions of psychological distress in which causal factors are associated with elements of the work environment. These disorders impose substantial economic and social burdens on affected workers and are frequently associated with alcohol consumption and the use of other psychoactive substances.

**Objectives:**

To conduct an epidemiological analysis of reported cases of work-related mental disorders in Brazil between 2012 and 2021, investigating their association with alcohol and other drug misuse.

**Methods:**

This analytical, quantitative study was based on notification data obtained from the Department of Informatics of the Brazilian Unified Health System. Sociodemographic characteristics, work-related conditions, and information on alcohol consumption and the use of other drugs were collected. Data were analyzed using statistical tests and multivariate analytical techniques.

**Results:**

Work-related mental disorders predominantly affected women, individuals with higher educational attainment, and those who self-identified as White or Brown. Alcohol consumption and the use of other drugs showed limited statistical significance as factors associated with the reported cases.

**Conclusions:**

Strategies aimed at improving working conditions, expanding access to psychological support, and preventing substance misuse are essential to address work-related mental disorders, a major public health concern in Brazil. Identifying risk factors associated with these disorders may inform more effective interventions and promote improved work organization, thereby reducing their impact on the health of the working population and on the national economy.

## INTRODUCTION

Work-related mental disorders (WRMDs) are defined as conditions of psychological distress that may present with diverse clinical manifestations but share a common feature: their causal factors are associated with elements of the work environment, whether inherent to work organization or resulting from occupational exposure to toxic agents [^1^]. Under Brazilian Law, WRMDs are classified as notifiable conditions, and health professionals are required to identify and report new cases on a weekly basis [^2^].

Considering the broader spectrum of mental and behavioral disorders, a marked global increase in their occurrence has been observed since the second half of the twentieth century, making them the second leading cause among the ten principal contributors to years lived with disability worldwide [^3^,^4^]. The etiology of this phenomenon is multifactorial, but accumulating evidence indicates that occupational activities play a central role in the development of these disorders. Work establishes a direct relationship between physical and psychological dimensions, and when this balance is disrupted, it may lead to mental illness, which is estimated to affect 17.6% of workers worldwide [^3^-^5^].

Increasing attention has been directed toward work-related mental health, particularly regarding how occupational activities influence workers’ behavior, lifestyle habits, and interpersonal relationships [^5^]. These interrelated factors appear to contribute to a concerning scenario: between 2003 and 2019, the average incidence of work-related mental and behavioral disorders among contributors to Brazil’s General Social Security System increased by 9.65% annually [^6^]. In 2021, mental health disorders were the third most common cause of temporary work disability in Brazil, accounting for 10% of expenditures on social security benefits nationwide [^7^].

These findings underscore the substantial economic burden associated with these conditions. In Europe, the combined costs of treatment, lost workdays, and rehabilitation are estimated at €617 billion annually. In Brazil, expenditures related to these disorders are estimated to account for 7.1% of all newly granted sickness benefits provided by the Brazilian National Institute of Social Security [^4^,^8^]. Although this figure represents an estimate, it highlights the considerable financial burden imposed by WRMDs on the Brazilian economy and reinforces the need for a more comprehensive understanding of their determinants, as well as the implementation of targeted prevention and intervention strategies [^4^].

Among the factors associated with the onset of these disorders are characteristics intrinsic to the employment relationship, including long working hours, whether or not accompanied by low wages, exposure to occupational hazards, disruption of sleep-wake patterns, conflictual interpersonal relationships in the workplace, and inadequate working conditions. The interaction of these factors may facilitate the progression from chronic physical strain to psychological distress [^5^,^9^]. In turn, this context may encourage the use and/or misuse of substances such as caffeine, tobacco, alcohol, psychotropic medications, and other drugs as coping strategies to alleviate occupational stress and frustration. Indeed, working conditions and the emotional responses they generate are recognized as important determinants of individual behaviors and lifestyle habits [^3^,^10^].

Excessive consumption of these substances may, in some cases, exacerbate preexisting emotional disorders and contribute to the use of antidepressants and stimulant medications, both of which also carry a risk of dependence. This cycle may be further intensified by the stigma surrounding mental disorders, making it more difficult for affected individuals to return to the workforce while impairing interpersonal relationships and family functioning. Consequently, demand for health care services and government expenditures on the care of this population increase substantially [^3^,^10^].

Accordingly, the present study aimed to conduct an epidemiological analysis of reported cases of WRMDs in Brazil between 2012 and 2021, with particular emphasis on their association with alcohol and other drug misuse, including tobacco use, psychoactive drug use, and psychotropic medication use. In addition, the study sought to characterize the epidemiological profile of affected workers and identify risk factors associated with the development of these disorders to inform future preventive and intervention strategies.

## METHODS

This analytical study employed a quantitative approach based on notifications of WRMDs obtained from the Department of Informatics of the Brazilian Unified Health System (DATASUS). The study population comprised all reported cases of WRMDs recorded in Brazil between 2012 and 2021. Cases with missing information on sociodemographic characteristics, working conditions, or habits related to alcohol and other drug use were excluded from the analysis.

Data were retrieved through the DATASUS File Transfer System using information recorded in the Brazilian Ministry of Health’s WRMD report forms. The following variables were collected: sex, race/skin color, educational attainment, alcohol use, psychoactive drug use, psychotropic medication use, smoking status, presence of other workers with the same disorder in the workplace, clinical management, and case outcome.

Data management, processing, and statistical analyses were performed using R version 4.4.1. Initially, a univariate analysis was conducted to assess data consistency and provide descriptive characterization of the study variables. Chi-square tests were subsequently applied to identify associations between variables, and statistical significance in these analyses served as the criterion for inclusion in the multivariate analyses. Multiple correspondence analysis (MCA) was then performed, incorporating all variables that demonstrated evidence of association with at least one other variable. Subsequently, the MCA-derived factor scores were used as input for a hierarchical clustering algorithm based on Euclidean distances to identify factors associated with a higher likelihood of alcohol and other drug use.

In a second stage, a count regression model was fitted, using the total number of reported cases as the dependent variable and the remaining study variables as independent variables. The suitability of data for Poisson or negative binomial regression models was assessed using the Cameron & Trivedi test [^11^] and the likelihood ratio test, adopting a 95% confidence level.

This study was conducted using a publicly available, open-access secondary database that contained no personal identifiers or information capable of identifying individual participants. Accordingly, approval by a research ethics committee was not required, in accordance with Resolution No. 510 of April 7, 2016, of the Brazilian National Health Council [^12^].

## RESULTS

The descriptive characteristics of the study sample, according to its sociodemographic and epidemiological distribution, are presented in Table 1, which also reports the monthly means, corresponding confidence intervals, and relative frequencies.

**Table 1.** Distribution of reported cases of WRMDs in Brazil between 2012 and 2021, according to sociodemographic and epidemiological characteristics

Variable	Category	Total	Monthly mean	95%CI	Relative total (%)
Sex	Female	10,925	70.03	5.80	63.66
	Male	6,236	39.97	2.81	36.34
Race/skin color	White	7,504	48.10	3.68	43.73
	Black	1,092	7.00	0.73	6.36
	Asian	153	0.98	0.21	0.89
	Brown	4,875	31.25	2.48	28.41
	Indigenous	44	0.28	0.09	0.26
Educational attainment	Illiterate	68	0.44	0.11	0.40
	Incomplete primary education (1st-4th grade)	287	1.84	0.26	1.67
	Incomplete primary education (4th grade)	220	1.41	0.20	1.28
	Incomplete primary education (5th-8th grade)	632	4.05	0.40	3.68
	Complete primary education	699	4.48	0.46	4.07
	Incomplete secondary education	838	5.37	0.52	4.88
	Complete secondary education	5,189	33.26	2.53	30.24
	Incomplete higher education	1,099	7.04	0.70	6.40
	Complete higher education	4,991	31.99	3.08	29.08
Alcohol use	Yes	1,021	6.54	0.67	5.95
	No	9,599	61.53	3.60	55.93
Psychotropic medication use	Yes	5,154	33.04	2.54	30.03
	No	5,873	37.65	2.35	34.22
Psychoactive drug use	Yes	1,341	8.60	1.26	7.81
	No	8,994	57.65	3.67	52.41
Smoking	Yes	841	5.39	0.47	4.90
	No	9,317	59.72	3.70	54.29
Former smoker	Yes	237	1.52	0.19	1.38
Removal from the mentally stressful situation	Yes	7,903	50.66	3.16	46.05
	No	5,150	33.01	3.27	30.01
Implementation of personal protective measures	Yes	1,456	9.33	1.24	8.48
	No	10,228	65.56	4.82	59.60
Changes in work organization	Yes	1,444	9.26	0.97	8.41
	No	10,314	66.12	4.73	60.10
No intervention implemented	Yes	1,079	6.92	0.80	6.29
	No	7,921	50.78	4.28	46.16
Implementation of collective protective measures	Yes	403	2.58	0.47	2.35
	No	11,112	71.23	5.05	64.75
Removal from the workplace	Yes	8,780	56.28	4.25	51.16
	No	4,112	26.36	2.23	23.96
Other workers with the same condition in the workplace	Yes	5,523	35.40	3.02	32.18
	No	2,916	18.69	1.60	16.99
Referral to a CAPS	Yes	9,134	58.55	3.84	53.23
	No	3,518	22.55	1.95	20.50

CAPS = Psychosocial Care Center; WRMDs = work-related mental disorders.

The Cameron & Trivedi test [^11^] indicated evidence of overdispersion in the analyzed data (t = −2.3699; p = 0.01903). Therefore, a Poisson-gamma regression model for count data was fitted.

**Figure 1.**
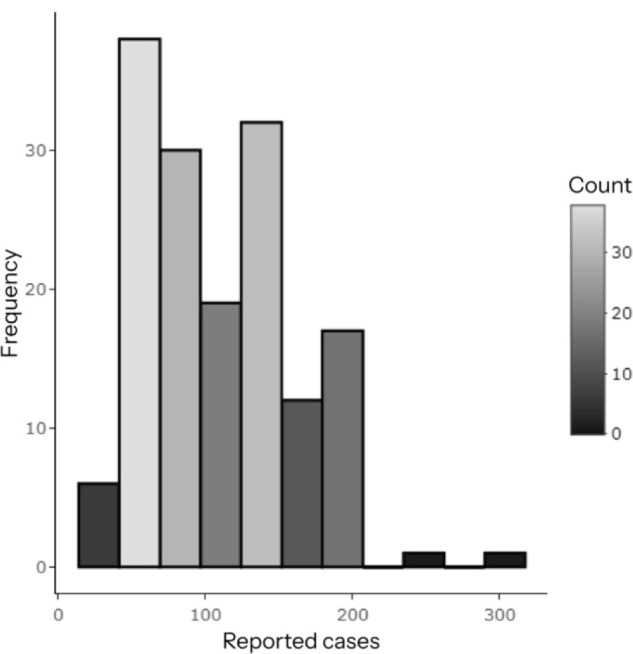
Distribution of the number of reported cases of work-related mental disorders.

The distribution of the dependent variable used in the model is presented in Figure 1.

The estimated coefficients for the independent variables, together with their corresponding standard errors, levels of statistical significance, and model fit statistics, are presented in Table 2.

**Table 2.** Poisson-gamma regression model for reported cases of WRMDs in Brazil between 2012 and 2021

Variable	Category	Coefficient	Standard error	z-value	p-value
Intercept		3.61×10^3^	3.42×10^1^	105.510	< 2×10^−16*^
Sex	Female	6.98	2.11	3.309	0.000935^*^
Race/skin color	White	3.88×10^−1^	1.67	0.233	0.815860
	Black	1.03	3.29	0.314	0.753569
	Asian	−2.62	7.39	−0.355	0.722447
	Brown	1.86	1.37	1.357	0.174914
	Indigenous	−5.78×10^1^	1.89×10^1^	−3.057	0.002237^†^
Educational attainment	Illiterate	3.90×10^1^	1.28×10^1^	3.046	0.002315^†^
	Incomplete primary education (1st-4th grade)	−8.15	7.15	−1.139	0.254692
	Incomplete primary education (4th grade)	3.26	7.88	0.413	0.679540
	Incomplete primary education (5th-8th grade)	−4.63	5.19	−0.892	0.372182
	Complete primary education	2.54×10^−2^	4.22	0.006	0.995193
	Incomplete secondary education	−5.89	3.46	−1.703	0.088514^‡^
	Complete secondary education	−1.41	2.08	−0.681	0.495687
	Incomplete higher education	5.22	3.60	1.453	0.146251
	Complete higher education	2.95	2.04	1.447	0.147946
Alcohol use		1.01	4.84	0.209	0.834487
Psychotropic medication use		−2.17	3.16	−0.688	0.491622
Psychoactive drug use		−2.37×10^−1^	5.36	−0.044	0.964787
Smoking		−3.99	5.31	−0.752	0.451765
Former smoker		6.02	7.89	0.762	0.445889
Removal from the mentally stressful situation		−1.43	2.68	−0.534	0.593486
Implementation of personal protective measures		7.78	6.59	1.182	0.237351
Changes in work organization		1.39	6.24	0.222	0.824323
No intervention implemented		5.10	3.02	1.690	0.091063^‡^
Implementation of collective protective measures		−8.07	7.92	−1.019	0.308125
Removal from the workplace		4.08	2.69	1.517	0.129315
Other workers with the same condition in the workplace		−3.57	1.07	−3.319	0.000905^*^
Referral to a CAPS		2.80	1.62	1.728	0.084049^‡^
Model fit statistics					Value
AIC					1,202.200
Theta					2,075,507
2 × log-likelihood					−1,112.246

AIC = Akaike information criterion; CAPS = Psychosocial Care Center; WRMDs = work-related mental disorders.

* p < 0.001

† p < 0.010

‡ p < 0.100

**Figure 2.**
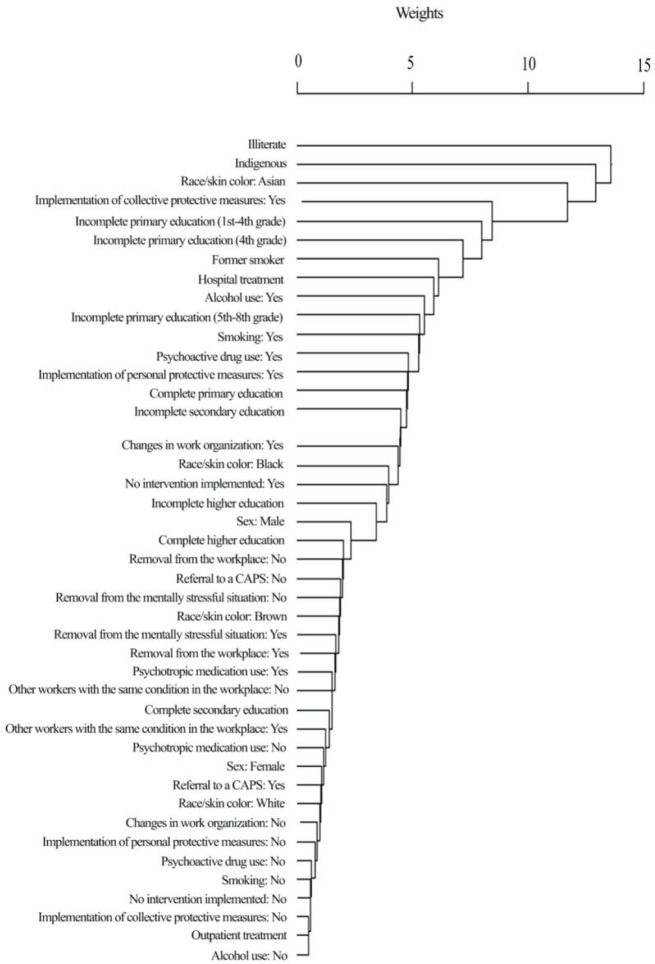
Dendrogram of the hierarchical clustering of variable categories derived from multiple correspondence analysis.

To further investigate the relationships among the independent variables included in the model, a MCA was performed. Subsequently, to account for the total inertia explained by the system, hierarchical clustering was applied to the MCA-derived factor scores. Figure 2 displays the association structure among the independent variables analyzed.

CAPS = Psychosocial Care Center.

## DISCUSSION

WRMDs represent a major public health challenge in Brazil, and the strategies currently in place, including the National Network for Comprehensive Workers’ Health Care, remain insufficient to adequately address this issue. In this context, the present study sought to identify risk factors associated with the occurrence of WRMDs and to characterize the epidemiological profile of affected workers, with the aim of informing strategies to improve work organization and strengthen psychological support for the workforce [^3^].

With regard to sex, women accounted for the majority of reported cases (63.66%). Interpretation of the coefficient associated with this variable in the Poisson-gamma model (Table 2) indicated a positive effect of female sex on the occurrence of WRMDs. Several factors may contribute to this finding. Women are generally more likely to seek health care services, thereby facilitating diagnosis and increasing the number of reported cases within this group [^13^]. In addition, historically assigned gender roles play a substantial role, as many women are exposed to a double workload by balancing paid employment with domestic and family responsibilities, a situation that increases both fatigue and emotional stress [^5^]. This burden is further compounded by persistent gender-related vulnerabilities, including the high prevalence of workplace moral and sexual harassment and persistent wage disparities relative to men, factors that make labor market participation more challenging and potentially more detrimental to mental health [^13^].

With regard to educational attainment, 30.24% of cases occurred among individuals who had completed secondary education, whereas 29.08% were reported among those with a completed higher education degree. Interpretation of the coefficients estimated by the Poisson-gamma model (Table 2) further demonstrated that both completed and incomplete higher education were positively associated with the occurrence of WRMDs. This finding may reflect the greater participation of highly educated individuals in the labor market, particularly in occupations characterized by high cognitive demands, elevated performance expectations, and substantial workloads, all of which may contribute to the higher number of reported cases observed in this group [^13^].

Analysis of race/skin color showed that the largest proportion of reported cases occurred among White (43.73%) and Brown (28.41%) workers, whereas only 6.36% of reported cases involved Black workers. Furthermore, interpretation of the coefficient associated with this variable in the Poisson-gamma model (Table 2) indicated that Brown race/skin color exhibited the strongest positive association with the occurrence of WRMDs.

The historical development of the Brazilian labor market has produced a context in which the Black population experiences disproportionately higher rates of unemployment and precarious working conditions than the White population, a phenomenon described as the racial division of labor [^14^-^16^]. Inequitable access to educational opportunities also represents a major barrier to the integration of Black individuals into the labor market [^16^]. Moreover, even among individuals with comparable educational attainment, Black workers generally receive lower wages than their White counterparts [^14^]. Considering these structural factors, together with the positive association between higher educational attainment and the occurrence of WRMDs observed in the present study, the limited access of the Black population to professional qualifications and formal employment may partially explain their lower representation among reported cases. However, this finding should not be interpreted as evidence of lower occupational vulnerability among Black workers or as suggesting that structural inequalities protect against psychological distress or work-related mental illness.

With regard to substance use, 5.95% of workers reported alcohol consumption, and 7.81% reported the use of psychoactive drugs. The Poisson-gamma model (Table 2) showed a positive coefficient for the association between WRMDs and alcohol use; however, this association was not statistically significant (p = 0.834487). According to the literature, occupational settings characterized by high levels of responsibility, intense job demands, inadequate working conditions, prolonged working hours, and insufficient managerial support may encourage alcohol consumption as a coping strategy for occupational stress [^4^,^5^]. Although these factors might suggest a stronger association between alcohol use and WRMDs, this hypothesis was not supported by the findings of the present study.

The use of these substances as a strategy for coping with the aforementioned occupational stressors may contribute to the development of substance dependence that is difficult to manage, with important consequences for both workers and the workplace. These consequences include reduced capacity to perform tasks and achieve work goals, job loss, interpersonal and family conflicts, neglect of social responsibilities, disruption of daily routines, neurological impairment with social repercussions, and the development of co-occurring mental disorders [^17^].

Psychotropic medication use was reported in 30.03% of reported cases. Despite this relatively high prevalence, the negative coefficient estimated by the Poisson-gamma model suggests a potential protective effect of this variable against the occurrence of WRMDs, although the association did not reach statistical significance (p = 0.491622). Assuming that these medications were prescribed as part of the pharmacological management of mental disorders, these findings are consistent with previous studies showing that, when appropriately prescribed and monitored, pharmacological treatment contributes to the control of acute symptoms and reduces work absenteeism [^18^,^19^]. Nevertheless, the effectiveness of psychotropic medications depends on the concurrent implementation of interventions targeting the social and occupational determinants underlying mental illness [^20^]. Notably, the database used in this study does not distinguish prescribed medication use from self-medication, representing a limitation of both the present study and the reporting forms used to record these cases.

An interesting finding emerged when examining the relationship between smoking cessation and the occurrence of WRMDs. Although former smokers accounted for only 1.38% of reported cases, the Poisson-gamma model (Table 2) revealed a relatively large positive coefficient for the association between smoking cessation and the outcome of interest, albeit without statistical significance (p = 0.445889). This finding may be related to evidence indicating that smokers with mental disorders generally exhibit greater nicotine dependence and experience more severe withdrawal symptoms during smoking cessation [^21^], as nicotine exerts mood-modulating effects that temporarily alleviate symptoms associated with these disorders [^22^]. Nevertheless, the scientific literature recommends that health professionals encourage smoking cessation, as evidence indicates that quitting does not worsen long-term mood and may in fact contribute to reductions in symptoms of depression and anxiety [^21^].

Analysis of the occupational context of the reported cases showed that, in 32.18% of reported cases, other workers in the same workplace had been affected by the same condition. This finding may reflect unfavorable organizational environments characterized by excessive working hours, intense performance demands, and inadequate institutional support for workers. Individuals exposed to hostile workplaces marked by high pressure and limited professional recognition are more likely to develop progressively more severe mental disorders over the course of their working lives [^5^].

Another important issue concerns the stigma surrounding mental health, which remains a taboo subject in many Brazilian workplaces [^4^]. Few managers receive specialized training or have access to qualified professionals capable of providing appropriate support, active listening, and guidance in addressing employees’ mental health needs. Furthermore, institutional strategies aimed at promoting psychological well-being remain limited, perpetuating a culture of silence surrounding mental health disorders in the workplace [^4^].

Regarding the interventions adopted, 51.16% of workers with WRMDs were removed from the workplace, and 53.23% were referred to a Psychosocial Care Center. Work absence imposes a substantial economic burden on the Brazilian social security system [^17^]. This finding is consistent with the fact that diseases classified under Chapter V (Mental and Behavioural Disorders) of the ICD-10 represent the third leading cause of social security benefit claims in Brazil. Although this social protection mechanism is essential, its coexistence with insufficient strategies for relapse prevention and sustainable return to work may contribute to prolonged and recurrent work absences, thereby increasing the economic burden of these disorders on the country [^23^].

Finally, analysis of the relationships among the independent variables included in the model showed that the dendrogram (Figure 2) identified one of the characteristic profiles of workers affected by WRMDs during the study period. This profile comprised women with high educational attainment who did not report alcohol use and for whom no specific intervention had been implemented following reporting of the disorder.

Further, alcohol use was not included in any of the characteristic profiles identified through the combination of MCA and hierarchical clustering. This result may be viewed as encouraging and reinforces the importance of maintaining public awareness campaigns on alcohol consumption, given its well-established individual and societal consequences, including impaired concentration, anxiety, neglect of responsibilities, and substance dependence [^24^].

Another finding that warrants attention is the absence of any intervention in a substantial proportion of reported cases. This observation suggests that, although policies and strategies aimed at promoting workers’ health, disease prevention, surveillance, and health care are in place, they remain insufficient to address the magnitude of the problem. This situation may be attributable to the lack of specific protocols for the management of WRMDs, a shortage of adequately trained professionals, the inherent complexity of identifying these disorders, and, above all, their limited visibility resulting from substantial underreporting within health information systems [^8^].

Importantly, this study is subject to limitations related to potential technical shortcomings in the reporting process for WRMDs and in the availability of the analyzed data, which may have influenced the findings.

## CONCLUSIONS

WRMDs represent a major public health concern, underscoring the need for continued attention to workers’ mental health. Despite advances achieved over recent decades, work-related psychological distress remains stigmatized and is frequently trivialized, contributing to the progression of increasingly severe conditions with substantial individual, social, and economic consequences.

By using MCA plus hierarchical clustering, the present study identified a characteristic profile of workers affected by WRMDs. This profile consisted predominantly of women with high educational attainment who did not report alcohol use and for whom no specific intervention had been implemented following report of the disorder.

These variables were also evaluated using a Poisson-gamma regression model to investigate their association with the outcome of interest. The findings showed that, although alcohol use was not part of the characteristic profile identified through the multivariate analysis, it exhibited a positive coefficient in the regression model, albeit without statistical significance. In contrast, psychotropic medication use, despite its high prevalence among reported cases, was associated with a negative coefficient for the outcome under investigation. Furthermore, female sex, Brown race/skin color, and higher educational attainment were positively associated with the occurrence of WRMDs.

These findings may help inform interventions aimed at improving work organization, strengthening psychological support for workers, and promoting rehabilitation strategies that facilitate a safe and sustainable return to work. Such measures are of considerable importance, as healthy work environments help protect against recurrent mental illness while preserving workers’ livelihoods, thereby alleviating, at least in part, the anxiety and uncertainty associated with work-related mental disorders.

## Data Availability

Upon publication, the data will be made available by the authors upon request
